# Application of Pyroelectric Sensors Based on PVDF Films for EPR Spectra Detection by Heat Release

**DOI:** 10.3390/s21248426

**Published:** 2021-12-17

**Authors:** Anatoly R. Melnikov, Samat B. Zikirin, Evgeny V. Kalneus, Vladimir I. Ivannikov, Yuri A. Grishin, Oleg A. Anisimov

**Affiliations:** Voevodsky Institute of Chemical Kinetics and Combustion SB RAS, 3 Institutskaya Str., 630090 Novosibirsk, Russia; samat@kinetics.nsc.ru (S.B.Z.); kalneus@kinetics.nsc.ru (E.V.K.); ivnvl@mail.ru (V.I.I.); grishin@kinetics.nsc.ru (Y.A.G.); anisimov@academ.org (O.A.A.)

**Keywords:** pyroelectric sensors, PVDF, electron paramagnetic resonance, reaction yield detected magnetic resonance, indirect detection of electron paramagnetic resonance, DPPH

## Abstract

Pyroelectrics are a wide class of materials that change their polarization when the system temperature varies. This effect is utilized for a number of different commercial and industrial applications ranging from simple thermal sensors and laser interferometers to water vapor harvesting. Electron paramagnetic resonance (EPR) spectroscopy is a powerful tool for studying the structure and dynamics of materials with unpaired electrons. Since heating accompanies a resonant change of the orientation of electron spins in an external magnetic field, pyroelectrics can be utilized as versatile detectors for so-called indirect detection of the EPR signal. In this work, we investigated three different types of PVDF (polyvinylidene difluoride) standard pyroelectric films with indium tin oxide, Cu/Ni, and Au coatings to determine their sensitivity for detecting EPR signals. All the films were shown to be able to detect the EPR spectra of about 1 μg of a standard stable free radical by heat release. A comparative study based on the calculation of the noise-equivalent power and specific detectivity from experimental spectra showed that the Au coated PVDF film is the most promising active element for measuring the EPR signal. Using the best achieved sensitivity, estimation is given whether this is sufficient for using a PVDF-based pyrodetector for indirectly detecting EPR spectra by recombination heat release or not.

## 1. Introduction

Pyroelectric and piezoelectric sensors made on the basis of flexible PVDF (polyvinylidene difluoride) films are successfully used in many fields of technology, medicine, and scientific research [1,2,3,4,5,6,7,8]. In particular, the pyroelectric properties of such films, which are previously poled in a strong electric field to enhance their performance, are used for the detection of radiation in various energy ranges, mainly in infrared and terahertz. These detectors can have different shapes and are cut from PVDF films with a thickness of several microns (most often 28 μm) that are metalized on both sides to register a pyroelectric signal. In order to measure the intensity of constant radiation flux, it should be modulated in intensity by a spatial beam modulator since the pyroelectric sensor responds to the change in temperature, not to absolute temperature. The modulated signal is amplified by a sensitive low-noise amplifier (LNA) and registered by a lock-in amplifier. Most often, a transimpedance operational amplifier circuit, operating in current mode, is used as LNA. Such a detection scheme is characterized by high sensitivity and good noise immunity, which makes it possible to register very weak heat fluxes and their change at the modulation frequency [9]. The choice of the correct parameters in the design of the circuit ensures its good stability [10].

Pyroelectric sensors can be used not only to measure weak external heat fluxes but also to study other processes leading to time-varying heating. Heating accompanies, for instance, the phenomenon of electron paramagnetic resonance (EPR), which consists in a resonant change of the orientation of electron spins in an external magnetic field under the action of a resonant microwave (MW) irradiation field [11]. This change in the spin orientation leads to the absorption of the energy of the MW field at the resonance by the sample containing free radicals. The dependence of the features of such absorption on the chemical structure of radicals and the kinetics of chemical reactions with their participation are the subject of EPR spectroscopy. Standard continuous wave (CW) EPR spectra are recorded by putting a sample into a MW irradiation field of constant frequency and sweeping the external magnetic field $B_{0}$ until the resonance condition is fulfilled. In the experimental setup, the MW field is built up in a resonator (typically a rectangular or cylindrical cavity), into which the sample (powder, solution, or a single crystal) is introduced. The resonator is critically coupled, which means that the incident power from the source of MW is completely absorbed by the resonator. Additional absorption by the sample during resonance leads to a detuning of the resonator and reflection of MW power that is recorded by a crystal detector located outside the resonator. Amplitude modulation of $B_{0}$ with a frequency of typically 100 kHz increases the signal-to-noise (S/N) ratio and is responsible for the derivative shape of the CW EPR spectra. The sensitivity of CW EPR spectroscopy allows detecting free radicals with an average amount of the order of 10^10^–10^11^ pieces per sample. The resonant change in the orientation of such a number of spins leads to power absorption in the range of 0.5–5 nW. The average energy consumed at the resonance as a result of the reorientation of one spin in the magnetic field of 350 mT at room temperature is about 10^−8^ eV.

Since the absorption of energy by the sample leads to its heating, this heating can be used to record EPR spectra instead of a standard measurement of the MW power reflected from the resonator. Such attempts have previously been conducted using bolometers [12], sensitive microphones [13], and pyroelectric detectors [14]. Despite encouraging results achieved in these works, the registration of EPR by heat release has not become widespread. The main reason is usually lower sensitivity compared to the standard CW EPR detection scheme. The situation is fundamentally different if the observation is carried out using recombining short-lived radical pairs instead of individual long-lived free radicals. In this case, the standard EPR method is often inapplicable because of the low average concentration of radicals due to their rapid recombination. Instead, a method of indirect detection of EPR spectra can be used. This method is called RYDMR (Reaction Yield Detected Magnetic Resonance) [15] and can be utilized, for example, to detect the EPR spectrum of paramagnetic particles that form spin-correlated radical pairs. In RYDMR, the resonant energy response of the sample depends on the collective spin multiplicity of the recombining pair and can be many times higher than the energy spent on the spin flip, reaching several eV. Frequently, the resealed energy is enough to electronically excite one of the molecules formed after recombination. As a result, the resonant change in the luminescence intensity of the excited states follows the EPR spectra of both radicals constituting the recombining pair. Due to the high energy response and close to unity difference in the population between collective spin sublevels of the correlated pair, optical registration (ODMR) of short-lived (0.1–1 μs) radical ion pairs gives a gain in sensitivity of the order of 10^7^–10^8^ in comparison with the standard CW EPR [16,17]. It should be mentioned that this gain is also provided by an extremely low level of intrinsic noise of photomultipliers [18], which are used if the signal is recorded by the intensity of the recombination luminescence. In addition to recombination luminescence, EPR spectra of paramagnetic particles participating in the recombination can be detected by the resonant change in the optical absorption intensity (ADMR) [19] or by the induced photoconductivity current (PCDMR, EDMR) [20]. In the last two decades, the use of indirect methods of EPR detection has been mainly associated with the study of photo-induced charge separation in organic semiconductor polymer systems and electroluminescent processes in organic semiconductors [21,22,23,24,25,26,27,28,29,30]. The active study of different semiconductors is mainly associated with their potential practical applications for the creation of organic solar cells and organic LED lighting sources.

The application fields of the RYDMR methods are not limited to the named systems and can be further expanded by the use of thermal detection. In such a case, RYDMR will become applicable to many photochemical processes, accompanied by the recombination of spin-correlated radical pairs. In this paper, we consider just such a possibility, in which recombination heat release is used for detection. In comparison with ODMR and EDMR, this method is more versatile since heat release is the most common consequence of any recombination, including spin-dependent. Heat release from the recombination of radical pairs of different multiplicities is expected to be different since the resulting products are typically distinct. Therefore, the resonant change in heat release should follow the EPR spectra of both radicals, constituting the recombining pair, in the same way as, for instance, in ODMR. A possible implementation of this detection type can significantly expand the range of applicability of RYDMR methods, extending them to objects in which there are no luminescence or charge carriers.

The possibility of detecting the recombination heat is mainly determined by the use of the most suitable detector (sensor). Such a sensor should have the following properties: (i) high sensitivity at room temperature, (ii) noise immunity, (iii) ability to operate inside a MW resonator, (iv) has a low weight to provide acceptable heating, (v) has a sufficiently large area for studying radical pairs generated in thin samples by external irradiation. Two types of modern film detectors have desirable properties. Firstly, VO_2_-based film bolometers, and secondly, commercially available PVDF films previously poled in a strong electric field and coated on both sides with a conductive layer. As was already mentioned, such films are widespread and usually used for the fabrication of various pyro- and piezoelectric sensors. Both types of detectors have approximately the same sensitivity to heating on the order of 10^8^ $\mathrm{cm}\sqrt{\mathrm{Hz}}W^{-1}$ [31,32]. We gave preference to the second option, as it allows the use of an individual sensor for each new sample due to its low cost and ease of fabrication. This work aims to investigate the behavior of pyroelectric sensors made of PVDF films under experimental conditions of EPR spectroscopy, to determine their sensitivity to the desired signal and various external interference of electromagnetic or acoustic nature, and to calculate their attainable signal-to-noise ratio. We study three different types of PVDF films with indium tin oxide (ITO), Cu/Ni, or Au coatings. Based on the results obtained, we discuss the possibility of using PVDF films for indirect EPR detection by recombination heat release.

## 2. Materials and Methods

**Figure 1:**
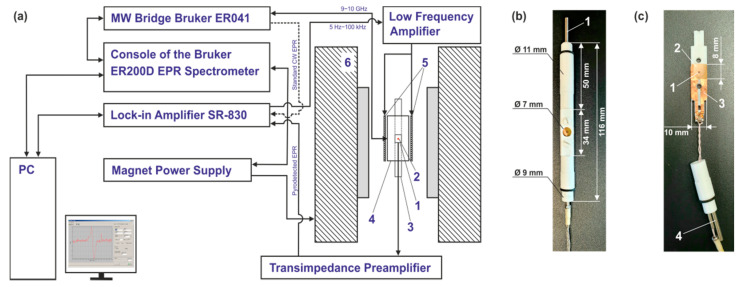
(**a**) General scheme of the experimental setup for recording pyrodetected or standard X-band CW EPR spectra. Numbers show: (1) PVDF film—an active element, (2) sample, (3) PTFE holder, (4) Radiopan RCX 661A X-band cylindrical resonator, (5) modulation coils, (6) magnet; (**b**) photograph of the PVDF based detector inside the PTFE holder. The number shows: (1) ventilation tube; (**c**) photograph of the disassembled detector. Numbers show: (1) DPPH sample, (2) Au coated PVDF film, (3) electrical contacts, (4) ventilation tube. The ventilation tube was made for future experiments and is currently not in use.

The active element of the sensor was based on commercial pyroelectric poled PVDF films manufactured by PolyK (PolyK Technologies, Philipsburg, PA, USA) with pyroelectric coefficient of about 30 μCm^−2^ K^−1^. The following 3 different films were used: (i) 28 μm thick film with 70 nm Cu + 10 nm Ni electrodes sputtered on both surfaces; sheet resistivity is equal to 1 Ω/sq, (ii) 28 µm thick film with an unspecified thickness of ITO electrodes sputtered on both surfaces; sheet resistivity is equal to 300 Ω/sq, (iii) 12 µm thick film with an unspecified thickness of Au electrodes sputtered on both surfaces; sheet resistivity is equal to 1 Ω/sq. For measurements, rectangular pieces of PVDF with a size of 10 × 9 mm were usually used. The sample under study (details are given below) was directly glued to the cut piece. The PVDF active elements were inserted into a special holder, which was placed in the resonator of ER 200D-SRC EPR (Bruker, Karlsruhe, Germany) spectrometer. An assembly consisting of a holder, an active element, and electrical contacts is hereinafter referred to as a pyrodetector or detector, and a rectangular piece of PDVF film is referred to as an active element. A block diagram of the experimental setup used and photographs of the pyrodetector in assembled and disassembled form are shown in Figure 1.

RCX 661A (Radiopan, Poznan, Poland) X-band cylindrical resonator with a working mode TM_110_ and unloaded Q-factor of about 5000 was used in all experiments. The active element of the detector was placed in the nodal plane of zero electric and maximum magnetic MW field of the resonator. In such a configuration, the PVDF film plane was tangential both to the direction of the external magnetic field $B_{0}$ and to the direction of the magnetic component of the MW field $B_{1}$. The described positioning of the active element in particular and the detector as a whole provided the minimum effect of both (i) the conductive coating of the PVDF film and (ii) the current-carrying electrodes (CCEs), made of the copper foil 50 μm thick (see number 3 in Figure 1c), on the Q-factor of the resonator. The contact of the copper CCEs with the conductive surfaces of the active element of the pyrodetector was provided by clamping them between 2 halves of the PTFE (polytetrafluoroethylene) holder using PTFE screws (see Figure 1b). CCEs were connected to the input of a home-built transimpedance preamplifier with a twisted pair cable about 0.4 m long, placed in an additional electromagnetic shield. The preamplifier output was connected to the input of SR-830 (Stanford Research Systems, Sunnyvale, CA, USA) lock-in amplifier using a standard 50 Ω cable. The lock-in amplifier was also used as a source of modulation frequency (from 1 Hz to 100 kHz), which was further amplified by a home-built low-frequency amplifier (see Figure 1a) and fed to the modulation coils of the resonator. The implemented registration scheme provided the possibility to switch from the PVDF signal detection mode to the standard one, in which a crystal detector was used. As a result, both types of EPR signals from the same sample can be registered without removing it from the resonator.

For efficient pyrodetection, the sample containing free radicals under study should be located in close proximity to an active element of the detector to give good thermal contact. Additionally, samples with the smallest possible weight give rise to a higher signal per unit mass, because they provide higher relative temperature changes under conditions of very weak heating caused by spin reorientation. Taking this into account, the proposed pyrodetector design is suitable for studying various thin-film polymers deposited directly on the surface of the detector’s active element. In such samples, free radicals can be generated by irradiation through a special hole in the resonator and in the top cover of the PTFE holder (see Figure 1). In this work, instead of generating active radical species by irradiation, we used a small amount of 1,1-diphenyl-2-picrylhydrazyl (DPPH) stable free radical. The CW EPR signal of DPPH at room temperature represents a single line with a width of about 0.18 mT. The simplicity of the spectrum and chemical stability allow using DPPH as a standard of the position and intensity of EPR signals. Thus, it can also be utilized to compare the sensitivity of different PVDF films. The samples for comparison were polycrystalline DPPH grains or powders, which were glued with epoxy to the central part of the active element of the detector. To determine the relative sensitivity of the active element used, it was necessary to know the number of radicals (spins) in the sample. If this amount was not too small, it could be accurately determined by weighing the DPPH powder on an analytical balance. However, if the weight of the sample was much less than 1 mg, it becomes difficult. To overcome this issue, we used the following comparative technique. First, a sample with a high amount of spins (about 1 mg of DPPH) was weighted and glued to the active element. Second, the standard CW EPR was measured for this sample in the absence of power saturation, and the absolute signal intensity was obtained. Finally, a sample with a significantly lower DPPH amount (visual control) was prepared, and the second step was repeated. The sought quantity can be determined by comparing the absolute intensities of the EPR signals of both samples, which for the given experimental conditions were directly proportional to the number of spins [33].

## 3. Results

### 3.1. Dependence of Pyrodetected and Standard CW EPR Signals on MW Power

**Figure 2:**
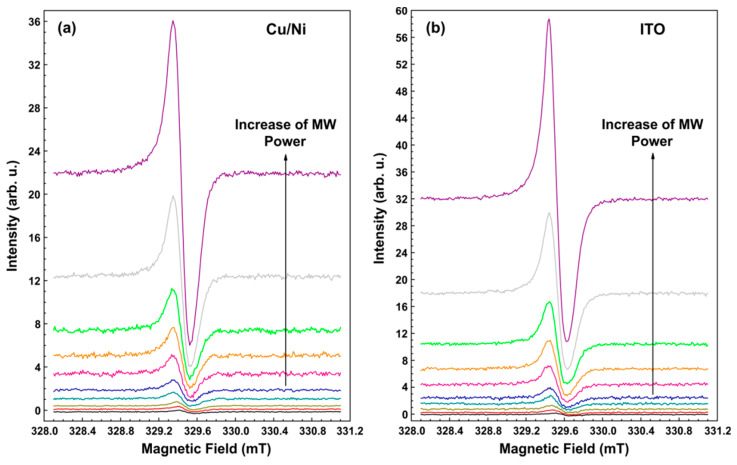
(**a**) EPR spectra of DPPH powder registered by a pyrodetector based on an active element with Cu/Ni coating at different MW power in the range of 0.2 mW (lower curve) to 40 mW (upper curve). The thickness of PVDF film is 28 μm. The amplitude of field modulation is 0.15 mT, the modulation frequency is 115 Hz, the time constant is 0.3 s, the temperature is 298 K. DPPH weight is 1.08 mg (about 1.6 × 10^18^ spins). The spectra are vertically shifted for better visibility. (**b**) The same with an ITO-coated active element. DPPH weight is 1.01 mg (about 1.5 × 10^18^ spins).

Figure 2 shows the dependence of the pyrodetected EPR signal of polycrystalline samples with a high DPPH amount (about 1 mg) on the power of the MW irradiation field. The active elements (28 μm thick) had the following types of coatings: (i) 70 nm Cu with a protective coating of 10 nm Ni and (ii) ITO. For both active elements used, a $B_{0}$ sweep of 3 mT was carried out for 80 s with a step of 0.011 mT (256 field points). As it follows from Figure 2, the lineshape of the detected spectra and the signal intensity are comparable for both active elements used. It means that the investigated Cu/Ni and ITO coatings do not significantly affect the properties of the resonator (see also Section 3.3), do not contribute to the recorded EPR spectrum, and do not distort the lineshape of the EPR signal.

**Figure 3:**
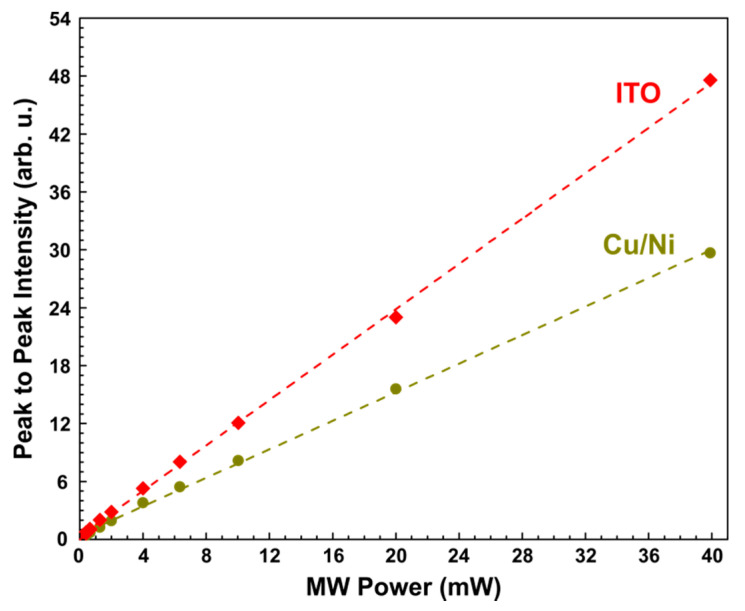
MW power dependency of the peak-to-peak intensity of shown in Figure 2 EPR signals. Red rhombuses and dark green circles correspond to the detection by Cu/Ni, or ITO-coated active elements, respectively. Red and dark green dashed lines show the best linear fit.

Figure 3 shows the dependence of the peak-to-peak signal intensity ($I_{pp}$) on the used MW power. $I_{pp}$ was obtained using data from Figure 2. As it can be seen from Figure 3, a good linear dependence is observed for both active elements, which is expected for pyrodetection in the absence of power saturation and in the case of uniform heating of the active element [12,14].

**Figure 4:**
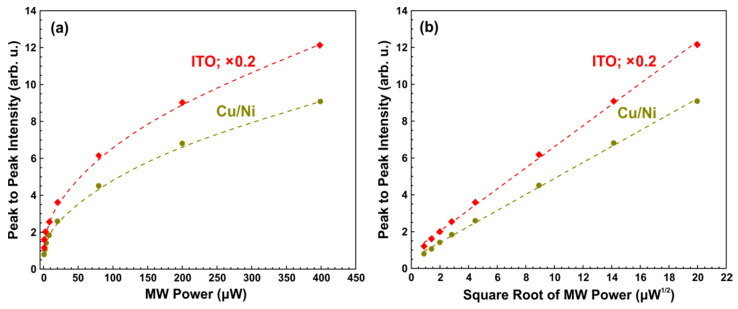
(**a**) MW power dependency of the peak-to-peak intensity of the standard CW EPR signals. Red rhombuses and dark green circles correspond to the DPPH powder, glued to Cu/Ni or ITO (multiplied by 0.2) active elements, respectively. The thickness of PVDF film is 28 μm. The amplitude of field modulation is 0.15 mT, the modulation frequency is 115 Hz, the time constant is 0.3 s, MW power is varied in the range of 0.8 μW to 0.4 mW, the temperature is 298 K. The assembly (active element with glued DPPH powder and PTFE holder) was the same as in the pyrodetected measurements (see Figure 2 and Figure 3). Dashed lines are guides to the eye. (**b**) The same as (**a**) in the linearized coordinates. Dashed lines show the best linear fit.

The same measurements were performed for the standard registration regime, in which a crystal detector was used. The assembly (active element with glued DPPH powder and PTFE holder) was the same as in the pyrodetected measurements. Figure 4 shows the obtained dependences of $I_{pp}$ on the MW power and their linearization. In this case, there is a square root dependence for both active elements, which is typical for standard CW EPR in the absence of power saturation [11].

### 3.2. Dependence of Pyrodetected EPR Signals on Modulation Frequency

**Figure 5:**
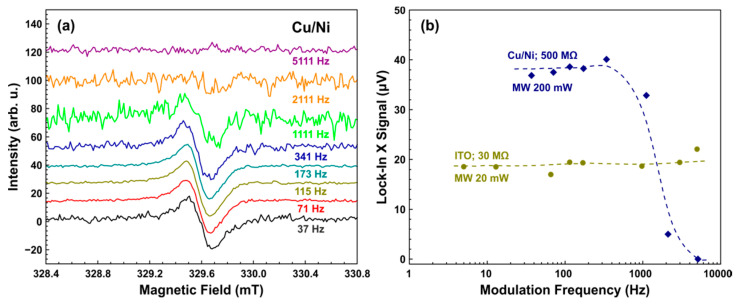
(**a**) EPR spectra of DPPH powder registered by the pyrodetector based on an active element with Cu/Ni coating at different MFs. The thickness of PVDF film is 28 μm. The amplitude of field modulation is 0.15 mT, modulation frequencies are in the range of 37 to 5111 Hz, the time constant is 0.3 s, MW power is 200 mW, the feedback resistor in the transimpedance preamplifier has the resistance ($R_{f}$) of 500 MΩ, the temperature is 298 K. DPPH weight is 2.2 μg (about 3 × 10^15^ spins). The spectra are vertically shifted for better visibility. (**b**) Modulation frequency dependency of the peak-to-peak intensity of the pyrodetected EPR signals for $R_{f}$ equals to 500 MΩ (Cu/Ni coating, data from (**a**), dark blue rhombuses) and 30 MΩ (ITO coating, DPPH weight is 1.01 mg, dark green circles, MW power is 20 mW, other experimental parameters are the same as for Cu/Ni coating). Dashed lines are guides to the eye.

As it was mentioned in the Introduction, the typical frequency of the amplitude modulation of $B_{0}$ is 100 kHz for the standard CW EPR. This value may be different for pyrodetection, where the kinetics characteristics of heat release and heat transfer can affect the signal level. Figure 5a shows the pyrodetected EPR spectra of a polycrystalline DPPH sample registered by Cu/Ni-coated active element at different modulation frequencies (MFs). The dependence of the obtained from the spectra peak-to-peak values on MF is given in Figure 5b. Two dependencies were measured, each with a different resistance ($R_{f}$) in the feedback loop of the preamplifier.

As can be seen from Figure 5b, at low MFs, the signal intensity does not depend on the frequency, which is typical for the current mode detection scheme of a “thermally thick” sensor [9]. The observed drop in intensity with increasing MF for the resistance of 500 MΩ was apparently due to a low-pass filter formed by the indicated resistance and stray capacitance in the feedback circuit [9]. Estimation of the stray capacitance from the presented dependence gives a value of the order of 0.3 pF, which seems to be reasonable. The assumption that the observed signal decay was associated with peculiarities of the used preamplifier circuit and not with any physical phenomena in the detector itself was confirmed by experiments with a resistance of 30 MΩ. They were carried out using an ITO-coated active element and a high DPPH amount of about 1 mg. A massive sample allows obtaining an acceptable signal level with a much lower MW power, which prevents excessive heating from a large number of spins in the sample. In this case (see Figure 5b), the signal intensity remains constant over the entire frequency range used, confirming that the decay was caused by the preamplifier circuit.

It should also be noted that an increase in the modulation frequency gives rise to a shift in the baseline level of the spectrum from zero for both active elements used in these experiments. The shift starts to be higher when the modulation frequency grows. Specially performed experiments have shown that it is caused by electrical interference on conductive parts of the detector. The source of interference is the modulation coils (see Figure 1) of the resonator. This shift did not affect the peak-to-peak intensity and shape of EPR signals obtained and was usually subtracted from the raw spectra.

### 3.3. Sensitivity of Different Active Elements of the Pyrodetector

**Figure 6:**
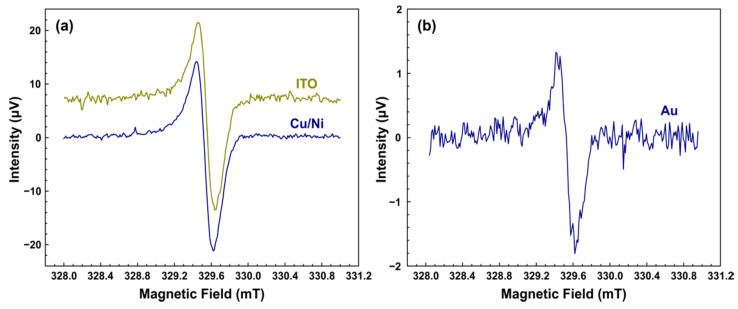
(**a**) EPR spectra of DPPH powder registered by the pyrodetector based on an active element with Cu/Ni (dark blue) or ITO coating (dark green, vertically shifted). The thickness of PVDF film is 28 μm. The amplitude of field modulation is 0.15 mT, the modulation frequency is 115 Hz, the time constant is 0.3 s, MW power is 200 mW, the temperature is 298 K. DPPH weight is 2.2 μg (about 3 × 10^15^ spins) and 2.8 μg (about 4 × 10^15^ spins) for Cu/Ni and ITO coating, respectively. Spectrum recording time is 400 s (Cu/Ni coating) and 1280 s (ITO). (**b**) The same as (**a**) for an active element with Au coating. The thickness of PVDF film is 12 μm. DPPH weight is 0.23 μg (about 3 × 10^14^ spins). Spectrum recording time is 2000 s.

The sensitivity of the pyrodetector was compared for three types of active elements: PVDF films with Cu/Ni, ITO, and Au coatings (for more details, see Section 2). Figure 6 shows the pyrodetected EPR spectra for all used active elements. The spectra were measured under identical experimental conditions, with the exception of the DPPH weight (0.1–3 μg) and the acquisition time (see Figure 6 caption). As can be seen from Figure 6, all the active elements allow detecting the EPR spectra of DPPH. The lower S/N ratio for the Au coating (see Figure 6b) was associated with an order of magnitude less DPPH in the sample. In the case of ITO and Cu/Ni coatings, the observed absolute signal intensities were similar for comparable amounts of DPPH. The main difference between the used active elements was their diverse influence on the properties of the resonator, in particular Q-factor. The ITO and Au coated films did not lead to a noticeable change in the resonator Q. At the same time, the use of the Cu/Ni coated film led to a decrease in Q by about 30%. Such a difference can be associated with the ferromagnetic properties of Ni.

To investigate the influence of applied MW power on the noise amplitude, special measurements were carried out far from EPR resonance with the MW power on and off. As it turned out, the noise level did not depend on the MW power for Cu/Ni and Au coated active elements. On the contrary, ITO-coated PVDF film showed a 4-fold increase in root mean square (RMS) noise when the MW power was varied from 0 to 200 mW. This behavior can be explained by the high sheet resistance of the ITO coating (300 Ω/sq) in comparison with Cu/Ni and Au that probably leads to additional nonresonant heating of the film by the MW irradiation field. Taking this into account, active elements with Cu/Ni and Au coatings are more promising for their use as pyrodetectors for EPR.

The sensitivity of a detector can be expressed in two different quantities: (i) noise-equivalent power, (ii) specific detectivity. Noise-equivalent power (NEP, $W/\sqrt{\mathrm{Hz}}$) is the minimum power required for an output S/N ratio of 1. Specific detectivity ($D^{*}$, $\mathrm{cm}\sqrt{\mathrm{Hz}}W^{-1}$) is related to the NEP value by Equation (1).

(1) $$D^{*}=\sqrt{A}/NEP,$$

where A is the sensor (PVDF film in our case) area.

Specific detectivity for a photodetector is a figure of merit used to characterize performance normalized per square root of the sensor’s area and frequency bandwidth (reciprocal of twice the integration time). Since specific detectivity is normalized, it is more suitable for comparing the detection efficiency of various active elements used in this work.

To calculate the thermal power P, supplied to the pyrodetector at the EPR resonance, Equation (2) can be used [12].

(2) $$P=\frac{sm_{0}N}{T_{1}},$$

where $m_{0}$ is the energy (per spin) spent on the resonant change in the sample magnetization, which is about 2.4 × 10^−27^ J at room temperature in a magnetic field of 0.35 T [12], N is the total number of spins in the sample, s is the so-called saturation parameter given by Equation (3), $T_{1}$ is spin-lattice relaxation time.

(3) $$s=\frac{B_{1}^{2}}{B_{1}^{2}+h^{2}},$$

where $B_{1}$ is the MW field strength in the rotating frame of reference, which is about 0.04 mT under the experimental conditions used, h is the saturation field equal to the value of $B_{1}$, at which the magnetization of the sample reaches half.

For a Lorentzian lineshape h can be found using Equation (4).

(4) $$h^{2}\;=\frac{1}{\gamma^{2}T_{1}T_{2}},$$

where γ is the gyromagnetic ratio for electron, $T_{1}$ and $T_{2}$ are the times of spin-lattice and spin-spin relaxation, respectively. $T_{1}$ for polycrystalline DPPH is 78 ns and is equal to $T_{2}$ [34].

Registered pyrodetected spectra had 256 magnetic field points (channels), corresponded to a $B_{0}$ sweep of 3 mT. The approximate number of points between the maximum and minimum of the recorded EPR spectra was 15.3, since, as it can be seen from Figure 6, the EPR linewidth of DPPH glued to PVDF film was about 0.18 mT. The power P absorbed by 2.2 µg DPPH sample placed on, for example, the Cu/Ni-coated active element at the resonance was, according to Equation (2), approximately 47 μW. Thus, the total power per point (channel) for this sample was about 3 μW (see Table 1).

The required for the calculation of NEP and $D^{*}$ S/N ratios were taken from the experimental spectra shown in Figure 6. In more detail, they were calculated as the ratio of the signal peak-to-peak amplitude and the RMS value of the noise. The resulting raw S/N ratio was recalculated on the total acquisition time per $B_{0}$ point to give a S/N ratio for an acquisition time of 1 s per point ${\left(S/N\right)}_{calc}\;$=$\;{\left(S/N\right)}_{exp}/\sqrt{t_{acq}}$. Finally, NEP was obtained as the ratio of the total power per point and the ${\left(S/N\right)}_{calc}$ of the corresponding coating.

The determined NEP and $D^{*}$ for all three active elements investigated are given in Table 1. As it can be seen from Table 1, the ITO-coated PVDF film has the lowest detectivity, while the Cu/Ni coating demonstrates the best performance. The significantly worse $D^{*}$ value for ITO coating was probably related to the previously mentioned dependence of the noise level on the MW power. In our opinion, the Au-coated PVDF film is the most promising active element for a special pyrodetector for measuring EPR signals by heat release. Although it showed slightly less sensitivity than the Cu/Ni-based detector, it had the least impact on the Q-factor of the resonator. As a result, high values of a $B_{1}$ field and, accordingly, high intensity of the EPR signal can be achieved with minimal parasitic heating of the sample by the nonresonant MW irradiation field.

The specific detectivity of the pyrodetectors obtained in this work was inferior to the previously achieved $D^{*}$ values for the best PVDF detectors. For instance, in ref. [32] for a PVDF film with a thickness of 9 μm, built into a readout microcircuit, a specific detectivity of $D^{*}$ equal to 4.4 × 10^8^ $\mathrm{cm}\sqrt{\mathrm{Hz}}W^{-1}$ was achieved. The theoretical sensitivity for pyrodetectors is further one to two orders of magnitude higher [35]. This indicates that the noise observed in our experiments limits the obtained sensitivity, and its source was not related to the internal noise of the detector. Possible causes could be external electromagnetic and acoustic interference. The presence of the acoustic component of the noise was confirmed by its increase in the presence of audible sounds in the room with the experimental setup, which was consistent with the well-known high piezoelectric sensitivity of PVDF films. Thus, suppression of electromagnetic and acoustic interference can probably increase the sensitivity of the pyrodetector to EPR signals.

## 4. Conclusions

In this work, we proposed a design and reported a performance study of a special pyrodetector for EPR signal detection by heat release. The active elements of the detector were fabricated using commercial poled PVDF films, coated on both sides with ITO, Cu/Ni, or Au. All the films were shown to be able to detect the EPR spectrum of DPPH by heat release. To determine the degree of influence of various factors on the active elements of the pyrodetector, the dependences of the observed EPR signals on the power of the MW irradiation field and the modulation frequency were measured. The ITO-coated PVDF film was found to have the lowest sensitivity, while the Cu/Ni and Au coating demonstrates good performance, according to the calculated specific detectivity $D^{*}$ and noise-equivalent power. Good sensitivity of the film with Cu/Ni coating is outweighed by a substantial decrease in the resonator Q-factor by about 30%, which is associated with the ferromagnetic properties of Ni. As a result, the Au-coated PVDF film was found to be the most promising active element for measuring the EPR signal by heat release since it has a high $D^{*}$ value and practically does not affect the Q-factor of the resonator.

Returning to the main motivation of this work, it would be instructive to estimate whether the currently achieved sensitivity is sufficient for using a PVDF-based pyrodetector for indirect detection of EPR spectra by recombination heat release or not. The minimum detectable signal power, as determined by the NEP value, is 230 nW or 1.6 × 10^12^ eVs^−1^. The number of radical pairs, the recombination of which leads to the appearance of EPR signal, in the most favorable case is about 1% of the total number of generated pairs. As was mentioned in the Introduction, energy of the order of 1 eV can be released during the recombination of a radical pair. Thus, the rate of their generation should be at least 1.6 × 10^14^ s^−1^ to be able to register them. If the lifetime of spin-correlated radical pairs generated at such a rate is, for instance, 1 μs, their average number in the sample will be 1.6 × 10^8^. Such a small amount is not available for detection using standard CW EPR, but is sufficient for registration with the pyrodetector. Taking into account that in photo- or radiation-chemical processes, an energy of about 10 eV is consumed to create one radical pair, the power of radiation absorbed in a thin sample deposited on the surface of the active element of a pyrodetector should be about 1.6 × 10^15^ eVs^−1^ or 0.2 mW. This value is reachable in typical photochemical experiments.

## Figures and Tables

**Table 1:** Several calculated characteristics of the three investigated PVDF films with different coatings.

Active Element Coating	$\mathrm{Power}\;\mathrm{per}\;B_{0}\;$ Point (μW)	${\left(S/N\right)}_{exp}$	$\mathrm{Acquisition}\;\mathrm{Time}\;t_{acq}$ $\;\mathrm{per}\;B_{0}\;$ Point (s)	${\left(S/N\right)}_{calc}\;{\;}^{a}$	$10^{9}\times NEP$ $\;(W/\sqrt{Hz}){\;}^{b}$	$10^{-6}\times D^{*}$ $(cm\sqrt{Hz}W^{-1}){\;}^{b}$
Cu/Ni	3.1	17.2	1.6	13.6	230	4.2
Au	0.3	3.0	7.8	1.1	300	3.2
ITO	4.0	7.1	5.0	3.2	1260	0.8

**^a^** S/N ratio for an acquisition time of 1 s per $B_{0}$ point; **^b^** since the samples usually occupied only the central part of the active element of the detector, the obtained sensitivity values are probably overestimated.

## Data Availability

Raw experimental data available upon request.
